# The 3.5 ångström X−ray structure of the human connexin26 gap junction channel is unlikely that of a fully open channel

**DOI:** 10.1186/1478-811X-11-15

**Published:** 2013-02-27

**Authors:** Francesco Zonta, Guido Polles, Maria Federica Sanasi, Mario Bortolozzi, Fabio Mammano

**Affiliations:** 1 Department of Physics and Astronomy “G. Galilei”, University of Padua, 35131, Padua, Italy; 2 Venetian Institute of Molecular Medicine, 35129, Padua, Italy; 3 International school for advanced studies (SISSA), 34136, Trieste, Italy; 4 CNR Institute of Neurosciences, Padua Section, 35131, Padua, Italy

**Keywords:** Connexin26, Calcein, Dual patch clamp, Fluorescence imaging, Umbrella sampling, Potential of mean force, Transition rate, Model

## Abstract

The permeability of gap junction channels to metabolites, and not simply to small inorganic ions, is likely to play an important role in development, physiology as well as in etiology of several diseases. Here, we combined dual patch clamp and fluorescence imaging techniques with molecular dynamics (MD) simulations to investigate the permeation of calcein, a relatively large fluorescent tracer (MW 622 Da) through homomeric gap junction channels formed by wild type human connexin26 (hCx26wt) protomers. Our experimental data indicate that the unitary flux of calcein driven by a 125 μM concentration difference is *J*_pore_ = 226 molecule/s per channel. In the light of Eyring transition state theory adapted for the liquid phase, this value corresponds to an energy barrier of ~20 *k*_*B*_*T* (where *k*_*B*_ is the Boltzmann constant and *T* is absolute temperature). The barrier predicted by our MD simulations, based on the 3.5 Å X–ray structural model of the hCx26wt gap junction channel, is ~45 *k*_*B*_*T*. The main contributions to the energetics of calcein permeation originated from the interaction between the permeating molecule and the charged aminoacids lining the channel pore. Assigning a fake zero total charge to the calcein molecule yielded a value for the barrier height compatible with the experimental data. These results can be accounted for by two different (although not mutually exclusive) hypotheses: (1) the X–ray model of the hCx26wt gap junction channel is not representative of a fully open state; (2) post translational modifications affecting the hCx26wt protein in our expression system differed from the modifications undergone by the proteins in the conditions used to obtain the crystal structure. Hypothesis (1) is compatible with data indicating that, only 10% or less of the channels forming a gap junction plaque are in the open state, and therefore the averaging procedure intrinsic in the generation of the crystal structure data more closely reflects that of a closed channel. Hypothesis (2) is compatible with recent mass spectrometry data and implies that the charge of several amino acid side chains may have been altered, thus modifying substantially the permeation properties of the channels in living cells.

## Background

Gap junction channels mediate communication between adjacent cells by allowing the passage of a variety of cytoplasmic molecules. They are formed by the head−to−head docking of two connexin protein hexamers, known as hemichannels or connexons, located in two adjacent cells [1]. Several studies showed that the permeation of cytoplasmic molecules through gap junction channels is fundamental in development and physiology, but also in the etiology of several diseases [2]. Second messengers, amino acids, nucleotides, glucose and its metabolites can permeate through at least some types of gap junction channels [3,4]. However, current understanding of the permeation properties and mechanisms is largely incomplete. Indeed, the unitary permeability of homomeric gap junction channels do not correlate well with ionic conductance and with presumptive pore sizes. The problem is exacerbated by the fact that gap junction channels in living cells can be formed by different connexin isoforms. Furthermore, most of the permeation properties of gap junction channels can, in principle, be dynamically regulated in response to external stimuli, such as voltage, pH or ionic concentrations [3,4].

The structure of connexin proteins and their assemblies was largely unknown until the publication of a model based on high resolution (3.5 Å) X–ray data of a hCx26wt channel [5]. The X–ray model permits to tackle issues left unresolved by previous models based on lower resolution data [6-9] such as the correct position of transmembrane helixes and the structure of extracellular regions. It also enables the study, by use of computational techniques, of ion permeation pathways [10,11] and the prediction of unknown structures (wild type human connexin30, hCx30wt) [11].

With its 226 amino acids, hCx26wt is one of the smallest member of the connexin family. Mutations of *GJB2*, the gene encoding hCx26wt, are implicated in both syndromic and nonsyndromic deafness [12]. The X–ray data indicate that hCx26wt comprises four transmembrane helixes (TM1, TM2, TM3 and TM4), which are connected by two extracellular loops (E1, E2) and one cytoplasm loop (CL) [5]. When assembled in hexamers, hCx26wt subunits create an aqueous pore in the plasma membrane, whose walls are formed by TM1 and TM2, plus the N–terminus (NT) that folds inside the pore at the cytoplasmic mouth of the channel. The mouth is created by the CL and part of the NT and hosts several positively charged residues. On the extracellular side, instead, hCx26wt presents an accumulation of negatively charged residues [5].

Here, we measured the unitary permeability of homomeric gap junction channels formed by hCx26wt to calcein, a widely used inorganic fluorescent tracer. We paralleled the experimental work with MD simulations [11] based on the 3.5 Å X–ray structure of hCx26wt [5]. Term of comparison between experiments and simulation is the transition rate of calcein through the channel, i.e. the number of calcein molecule that are able to traverse the channel per unit time. Our results indicate that the 3.5 Å X–ray structure of hCx26wt is unlikely that of fully open channel and suggest that permeation properties of the channel may be significantly affected by post–translational modification of critical residues lining the pore.

## Results and discussion

### Experimental determination of calcein transition rate

**Figure 1:**
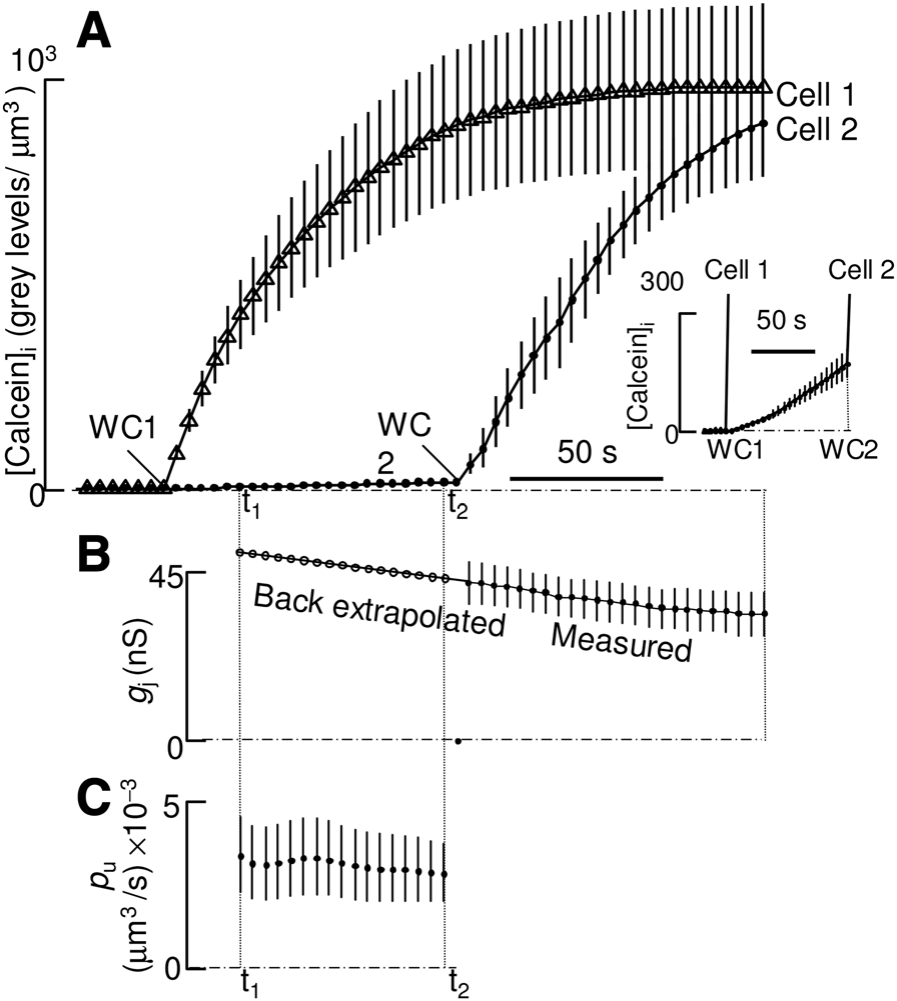
**Estimate of hCx26wt unitary permeability to calcein in pairs of transfected HeLa cells.** (**A**) Average fluorescence traces (*n* = 3) obtained by illuminating the field at *λ*_ex_ = 465 nm to observe calcein spread (emission wavelength *λ*_em_ = 520 nm); calcein (125 μM) was delivered to cell 1 under whole−cell recording conditions (WC1); ordinates are calcein concentration (see Methods); triangles correspond to cell 1, dots to cell 2; bars represent standard error of the mean (SEM). To better appreciate the rise in fluorescence in cell 2, the inset on the right shows a close − up view between WC1 and the time of achieving the whole–cell configuration in cell 2 (WC2). (**B**) The junctional conductance (*g*_*j*_) value required to estimate the single channel permeability *p*_u_ to calcein was back extrapolated from the time course of *g*_*j*_ after WC2. (**C**) The value of *p*_u_ = 3.0 ± 1.0 (mean ± SEM) was computed in the interval between times t_1_ and t_2_ where the signal–to–noise ratio was optimal.

We obtained an experimental estimate for the unitary flux of calcein using HeLa cells transiently transfected with cDNA encoding hCx26wt, as previously reported [13]. Calcein was delivered intracellularly, under whole−cell recording conditions, by passive diffusion out of a patch pipette filled with 125 μM of this dye. Transfer of calcein between HeLa cell pairs coupled by homomeric hCx26wt channels was monitored by wide−field fluorescence microscopy followed by direct measurements of cell volume by digital optical sectioning [14]. Based on the measurement of the unitary permeability *p*_u_ = (3.0 ± 1.0) × 10^−3^ μm^3^/s (mean ± standard error of the mean, SEM) presented in Figure 1, we estimate *J*_pore_ = *p*_u_ (*c*_1_*−c*_2_) = 226 ± 75 molecules/s per channel to be the unitary flux of calcein driven by a concentration difference *c*_1_*−c*_2_ = 125 μM (see Methods). A possible interpretation of this result is that, on average, a calcein molecule traverses a hCx26wt channel in a time τ_Exp_ = 1/226 s = 4.4 ms. We thus define the experimentally determined transition rate for calcein (with a concentration difference of 125 μM) as *k*_Exp_ = 1/ τ_Exp_ = 226 s^−1^.

### MD analysis of the permeation process

**Figure 2:**
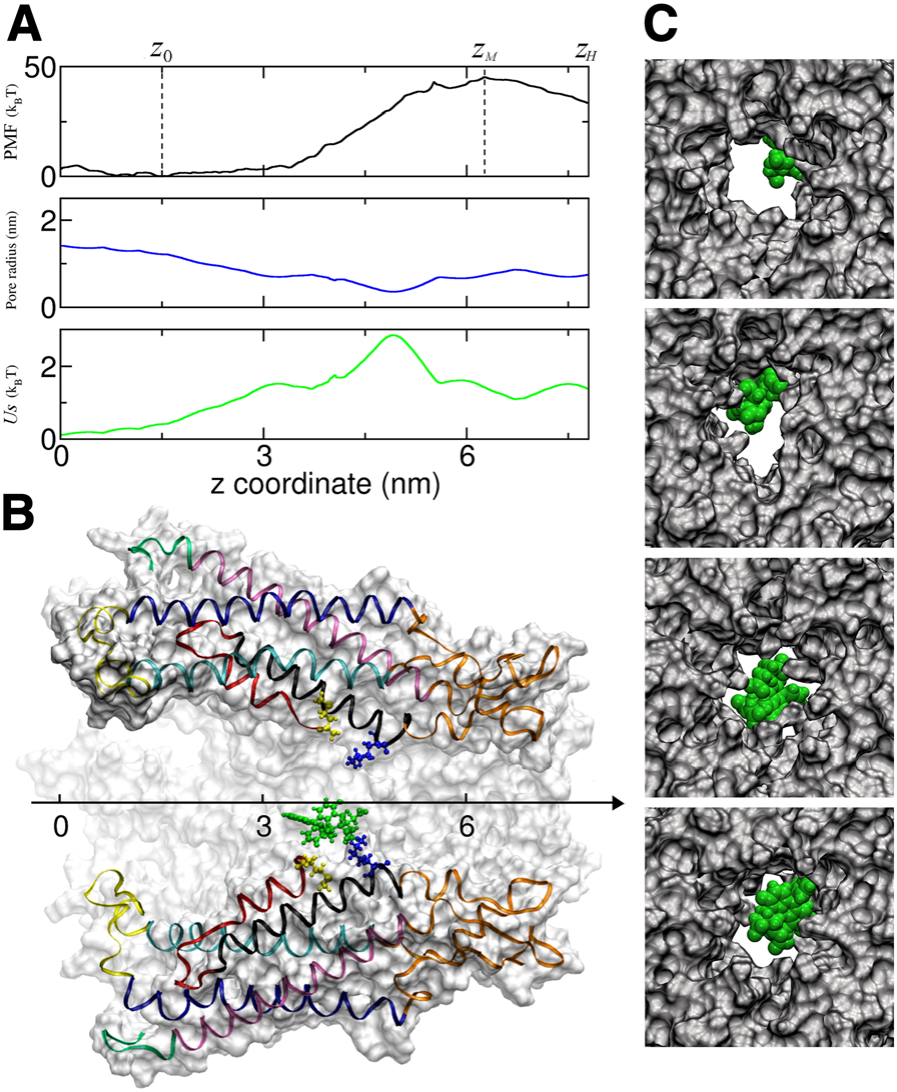
**MD simulation of the calcein permeation process.** (**A**) black trace: PMF of permeation through the hCx26wt hemichannel vs. axial position (z) with the calcein molecule in a fully charged state; blue trace: hemichannel pore radius green trace: entropic contribution to the PMF due to translational motion of calcein within the pore; note that this contribution is not sufficient to justify the high free energy barrier encountered by calcein (black trace). (**B**) Hemichannel section (side view) shown in register with the graphs of panel A; two connexin protomers are drawn in ribbon style; colors encode structural domains; a calcein molecule (green) and two residues that strongly interact with it (Met1, yellow, and Lys41, blue) are drawn in ball−and−stick style. (**C**) Views from the extracellular side of the hemichannel in four different umbrella sampling simulations.

Direct simulation of permeation of ions or molecules through a gap junction channel is beyond the current computational power due to the time scales involved (of the order of τ_Exp_, i.e. a few ms). For a system as large as the one we are examining (with > 2×10^5^ atoms) this exceeds by far the time window of state of the art MD simulations (0.1 to 1 μs). For this reason we used an indirect method based on the estimate of the free energy profile for the permeation process [15,16], which can be approximated by the potential of mean force (PMF) [17] for calcein permeation through a homomeric hCx26wt hemichannel (Figure 2A, top panel). Initially, we computed this PMF using the umbrella sampling technique [18] (see Methods) assuming that all carboxyl groups of calcein are deprotonated, as expected at neutral pH in the bulk (i.e. when calcein is well solvated and essentially isolated within the solvent). At axial coordinate *z*_*M*_ , the PMF in Figure 2A reaches a peak *W*_*c*_ = 45.2 *k*_*B*_T (the subscript *c* stands for “charged” calcein).

The calcein transition rate *k*_*c*_, estimated from the PMF profile as described in the Methods, is twelve orders of magnitude smaller than *k*_Exp_, meaning that no calcein molecule would ever traverse a homomeric hCx26wt gap junction channel with the structure predicted by the 3.5 Å X–ray data [5].

To find a rationale for the striking contrast between experimental results and MD model predictions we examined various possibilities. First, we noted that the channel pore narrows in the regions of fastest PMF increase (Figure 2A, middle panel), forcing calcein to interact with two positively charged residues, namely Lys41 and Met1 (Figure 2B). In our MD model, the latter has a protonated amino group because of its terminal position. Snapshots of calcein in four umbrella sampling windows spanning this critical region are presented in Figure 2C. A particle moving in a channel with variable radius experiences a net entropic force due to the variation of the accessible phase space in the various segments of the channel. According to Zwanzig theory [19], this force is equivalent to a potential

$$U_{S}\left(z\right)=-k_{B}T\;\ln\left(\frac{A\left(z\right)}{A\left(z_{0}\right)}\right)$$

where *A* is the section area of the channel, and *z*_0_ a reference position. Function *U*_*S*_(*z*) for the hCx26wt connexon is plotted in Figure 2A (bottom panel). Not only is the maximum value of *U*_*S*_(z) significantly smaller than *W*_*c*_, but *U*_*S*_(z) is also qualitatively different from the PMF profile. Based on this analysis we conclude that the entropic contribution to the PMF due to the variable radius of the pore is not the dominant factor and the channel is not closed from a purely entropic point of view.

**Figure 3:**
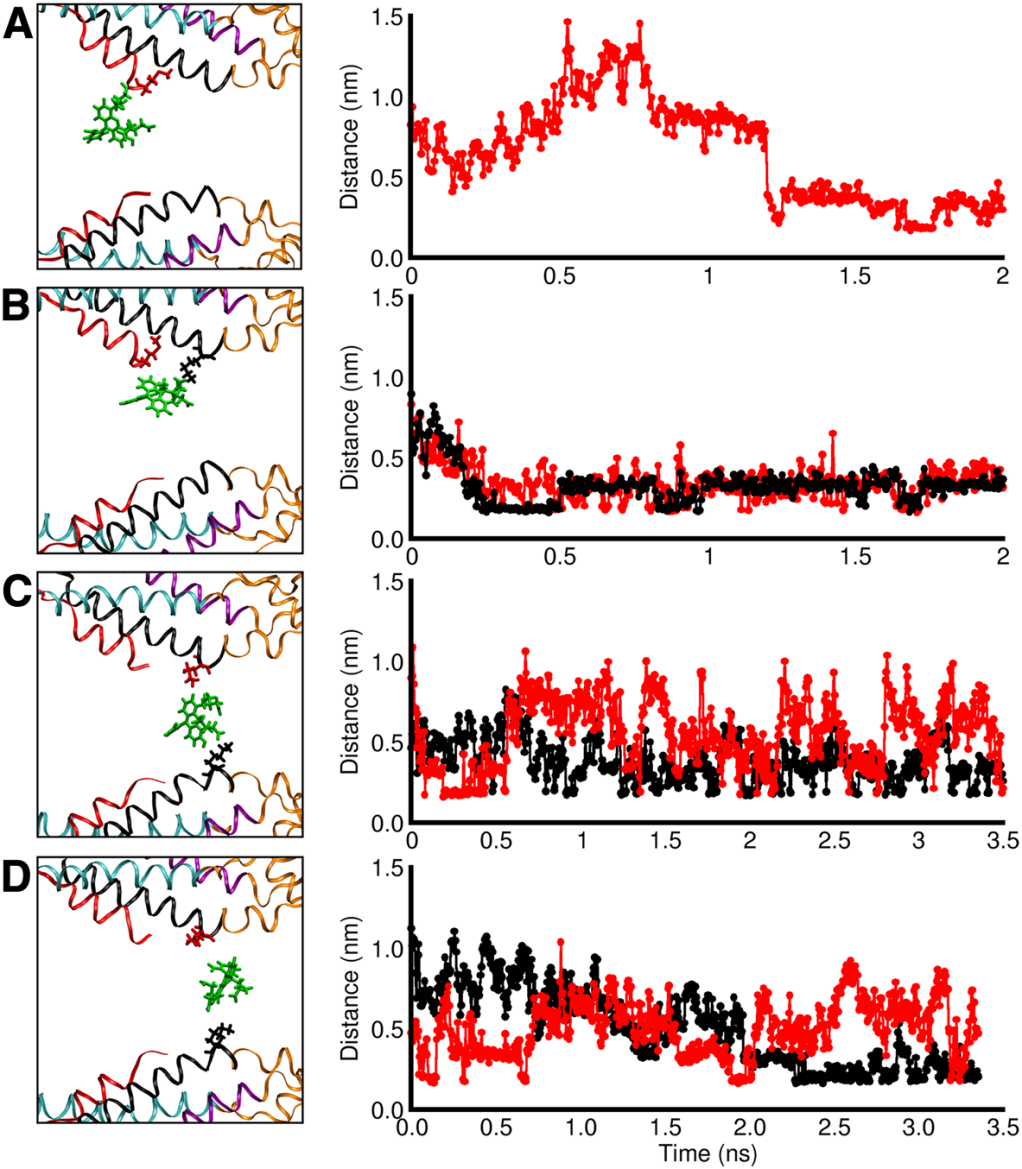
**Formation of salt bridges in different umbrella sampling windows.** Left panels (**A**−**D**) show side view representations of the calcein molecule in the channel pore; residues that interact with it are drawn in ball−and−stick style. Right panels (**A**−**D**) show time−course plots of the distance between one carboxy group of calcein and the amino group of interacting residues. (**A**) Calcein interacts with a Met1 alone. (**B**) Moving along the axis of the pore, calcein is found in a favorable position to interact both with Met1 and with Lys41. (**C, D**) After breaking the interaction with Met1, calcein interacts only with Lys41; in this zone, salt bridges are less stable due to calcein interacting simultaneously with Lys41 residues from different protomers.

Next we examined the issue of electrical charges. As noted above, in the course of our umbrella sampling simulations Met1 and Lys41 interacted with the negatively charged calcein molecule (Figure 2B). These interactions are further explored in Figure 3, showing that calcein carboxyl groups formed salt bridges with the amino groups of Met1 and Lys41. In the wider region of the narrowing pore, calcein formed bridges with Met1 alone (Figure 3A) or with both Met1 and Lys41 (Figure 3B); these bridges then remained stable for the rest of the dynamics. The region at TM1/E1 border barely accommodated one calcein molecule with a partial hydration shell. Here calcein interacted only with Lys41, which protruded into the pore (Figure 3C,D, left). However, due to the presence of six charged Lys41 side chains within this narrow region, the salt bridges were less stable, and tended to be exchanged between different protomers (Figure 3C,D, right). A calcein molecule needs to break and reform salt bridges to move within this region, and this explains the virtually impenetrable energy barrier that it encounters.

**Figure 4:**
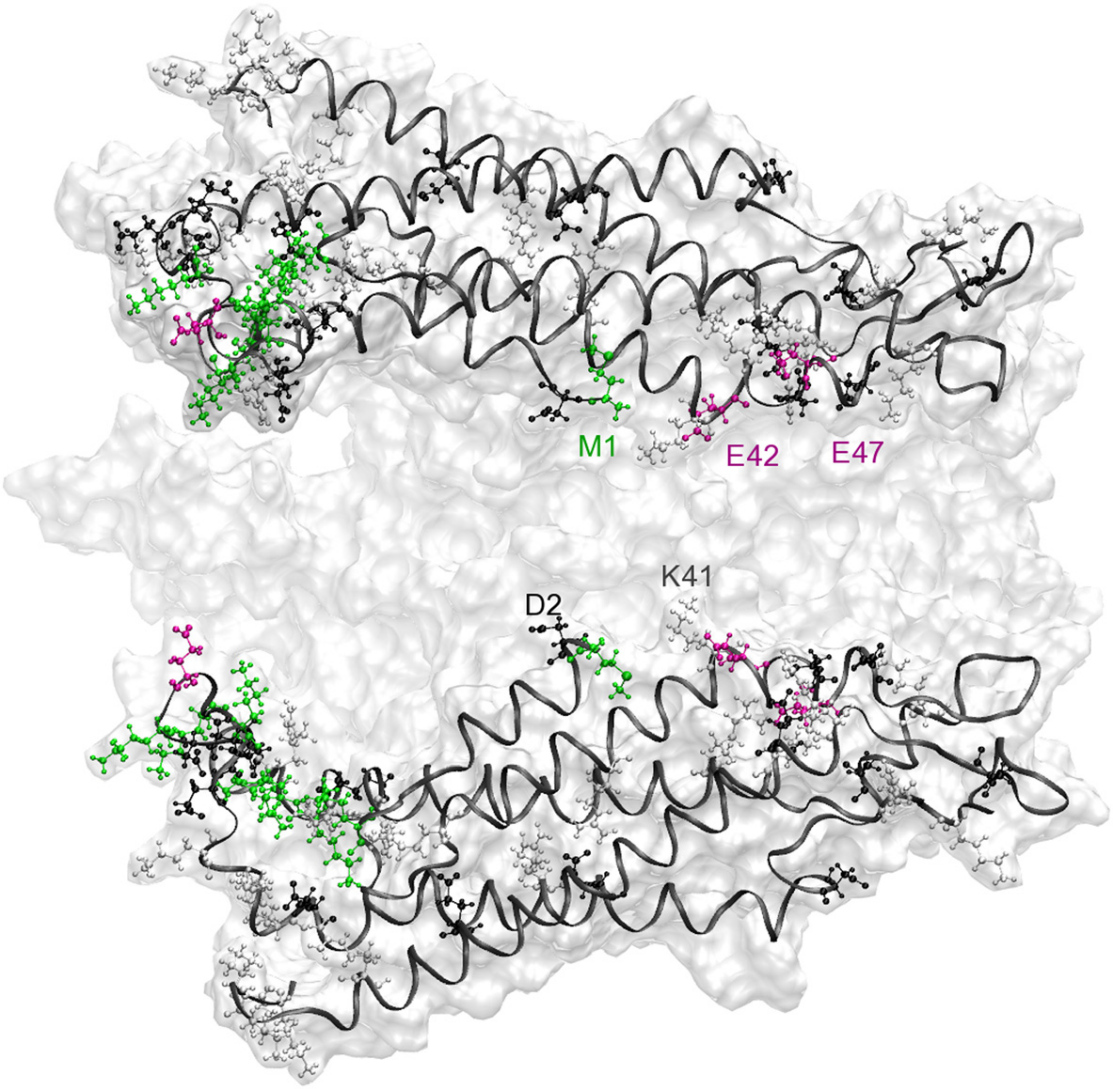
**Post–translational modifications candidates.** Amino acids which are candidates for post translational modifications, according to Ref. [20], and other charged amino acids are drawn in ball−and−stick style. Green: arginines and methionines that could be acetilated (global charge = 0); magenta: glutamic acids that could be gamma−carboxilated (charge = −2); white: arginines and lysines (charge = +1); black: aspartic acids and glutamic acids (charge = −1).

**Figure 5:**
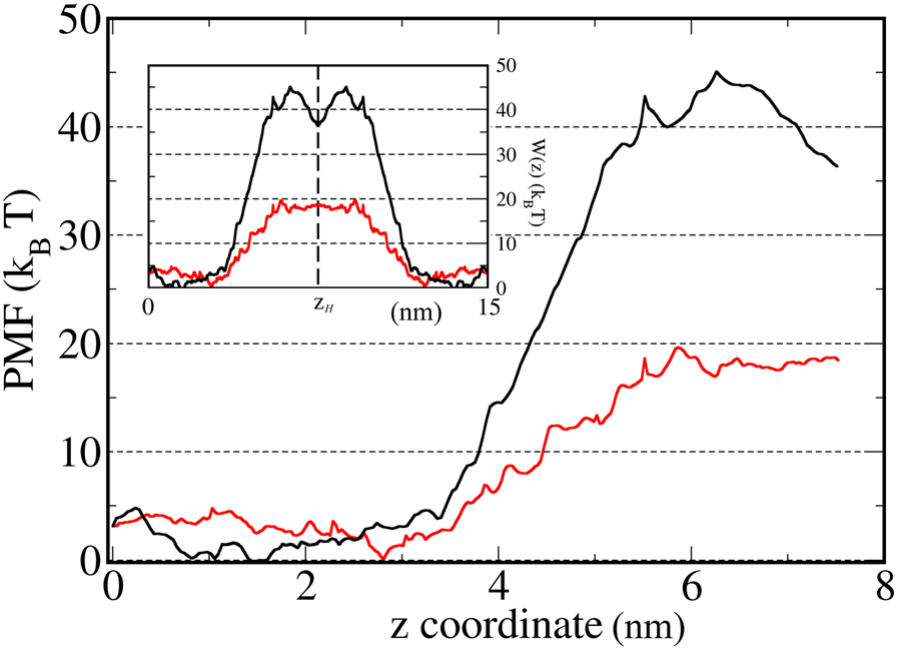
**Comparison of PMFs for the permeation of charged (black) and uncharged calcein (red).** The main graphs show PMF profiles for a hCx26wt hemichannel. Inset: corresponding free energy profiles, *W*(*z*), for a complete gap junction channel obtained by reflecting the hemichannel PMFs about a vertical axis through the *z*_*H*_ abscissa.

However, there is evidence that several charged residues of hCx26wt can be modified by post translational modifications (Figure 4), which may also depend on cell condition such metabolic stress [10]. In particular it was shown that Met1 and seven lysines are acetylated (K15, K102, K103, K105, K108, K112 and K116), whereas three glutamic acids are gamma–carboxilated (E42, E47 and E114) [20]. Due to the difficulties of testing these sites, which requires building a new channel model for each candidate (and combinations thereof), we decided to reverse the point of view and cancel electrostatic interactions by eliminating calcein charges. We then performed a second set of simulations with calcein in a fully protonated state (i.e. with zero total charge). The results in Figure 5 show a PMF with a greatly reduced peak *W*_*u*_ = 19.6 *k*_*B*_T (where the subscript *u* stands for “uncharged” calcein), which corresponds to a transition rate *k*_*u*_ = 352 s^−1^ (see Methods), in far better agreement with *k*_Exp_ = 226 ± 75 s^−1^.

## Conclusions

In this paper we measured the unitary flux of calcein through hCx26wt gap junction channels, and compared the experimentally determined value to that predicted by MD simulations based on the 3.5 Å X–ray structural data [5]. Term of comparison is the unitary transition rate, i.e. the number of calcein molecules that are able to transit trough a single channel per unit time. Simulations were performed with two different charge states for the calcein molecule. In the first case calcein had all the carboxyl groups deprotonated, as expected at physiological pH. In the other case, calcein was protonated and set to zero total charge. Our simulations indicate that a calcein molecule with a presumptive physiological charge is unable to traverse the channel due to the large energy barrier it faces (45.2 *k*_*B*_*T*). In contrast, the predicted transition rate for a calcein molecule with zero charge is compatible with the experimentally determined value.

Based on this analysis we conclude that the structural model of the hCx26wt channel derived from the 3.5 Å X–ray data [5] is not permeable to calcein (even after MD relaxation) and the blockade is essentially electrostatic. Our conclusion is in contrast with the proposal of Maeda et al. [5] that the model represents an open channel. This proposal was based on the facts that: (i) unlike the M34A mutant channel structure [21], there are no obvious obstructions along the pore of the hCx26wt channel; (ii) the crystallization conditions adopted by Maeda et al. are compatible with the formation of channels in the open state (neutral pH without aminosulphonate buffer or any divalent ions). The discrepancy highlighted in the present work can be explained as follows: (1) there is no way to guarantee that the open channel structure was preserved during the partial dehydration and crystallization procedures; (2) in a gap junction plaque, only 10% or less of the channels are in an open state [22-24].

The following considerations lend further support to this conclusion. Structure relaxation during our MD simulations (carried out in a realistic environment) resulted in a widening of the pore, particularly at the cytoplasmic mouth of the channel [11]. Even this wider pore is impermeable to charged calcein. Moreover, as mentioned in the results, the charges of several residues facing the pore may be altered by post translation modifications, which may differ in the mammalian (HeLa) cells used in our experiments with calcein and in the insect cells used by Maeda et al. in their crystallization study [5]. A recent study showed that modification of the charge of these residues is sufficient to recover the correct current−voltage (I−V) relationship and ionic conductance, with the main role played by Met1 [10]. Among these, Met1, Glu42 and Glu47 are crucially located in the region of steepest PMF increase. Our results indicate that charged Met1 and Lys41 play a crucial role in hindering calcein permeation. Note that Lys41 is not a candidate for acetylation based on Ref. [20]. Furthermore, acetylation of this residue would reduce significantly the diameter of the pore at its narrowest point, thus we consider it unlikely. In this scenario, gamma–carboxilation of Glu42 can be a fundamental determinant of channel permeability. Indeed, the carboxylated side chain of Glu42 is well poised for interacting with the amino group of Lys41. This interaction could stabilize the side chain of Lys41 (which instead appears rather mobile in our simulations) reducing the electrostatic potential felt by permeant molecules in the narrowest part of the channel, altogether favoring their transit. Further simulations are required to explore the influence of post translation modifications on the PMF of the channel not only for small inorganic ions but also for large permeant molecules.

## Methods

### Experimental methods

#### Dual whole cell patch–clamp recordings and fluorescence imaging

A detailed description of the theoretical and experimental framework for our permeability assays based on double patch–clamp and fluorescence imaging is provided in the Supplementary Methods of Ref. [13]. In brief, HeLa cells were co–transfected with a pcDNA3.1 expression vector carrying the coding region of hCx26wt and an additional expression vector carrying a cytosolic CFP to identify transfected cells. An isolated pair of cells was contacted in the cell–attach configuration by patch–clamp pipettes loaded with calcein (Invitrogen, C481) at 125 μM concentration. At time zero, the whole–cell configuration was established in cell 1 (WC1, Figure 1A), permitting the diffusion of calcein from cell 1 to cell 2 via overexpressed gap junction channels. The rise of fluorescence in cell 2, in addition to the estimates of the number *N*_pore_ of open channels and the volume *V*_2_ of cell 2, permitted us to derive the single channel permeability value *p*_u_ as

$$p_{u}=\frac{V_{2}}{N_{\mathrm{pore}}}\frac{\mathrm{dc}/\mathrm{dt}}{c_{1}-c_{2}}$$

where *c*_1_ is calcein concentration in cell 1 and *c*_2_ < *c*_1_ is concentration in cell 2. *N*_pore_ was derived by dividing the total junctional conductance *g*_j_ back extrapolated between WC1 at WC2 (Figure 1B) by the previously determined single channel conductance *γ* =115 pS [13].

### Numerical simulation methods

#### hCx26wt connexon MD model

The fully atomistic model used for the hCx26wt hemichannel was developed in our previous work [11]. Briefly, we completed the published structure [5], adding the atoms that were missing in the original structure, and then inserting the initial hCx26 connexon configuration in a hole opened in a pre–relaxed membrane bilayer of phospholipids (palmytol posphatidyl choline, POPC). The final membrane configuration comprises 493 phospholipids. The positive net charge of the hCx26 connexon was neutralized with 54 chloride ions; additional pairs of potassium and chloride ions were added to mimic a physiological ionic strength. The system was solvated with a total of 39189 water molecules.

#### Calcein parametrization

The calcein molecule parameters required by our Molecular Dynamics simulations are not present in any standard library. We parameterized it as described below in two different protonation states: (i) standard charge, as reported by the manufacturer (Invitrogen, C481); (ii) completely protonated, i.e. zero total charge. The initial guess of calcein coordinates was obtained using the GlycoBioChem PRODRG2 server and the JME Molecular Editor provided on the server [25], from the molecular structure provided by the manufacturer of the calcein moiety used in the experiments (Invitrogen). After this step we refined the coordinates and obtained the parametrization for GAFF force field of the two different protonation state of calcein using the Antechamber package [26].

#### Evalutation of PMFs by use of the umbrella sampling technique

The PMF [17] is an approximation of the free energy changes along one or more reaction coordinates. One of the most frequently used and effective methods for computing it is the umbrella sampling technique [18,27,28]. The starting model used for the umbrella sampling simulation of calcein transition through the channel was taken from our previous work [11]. Initial configurations for each window of umbrella sampling were extracted from a steered Molecular Dynamics trajectory of the calcein transition through a hCx26wt hemichannel. In this preliminary simulation, the calcein molecule was dragged through the pore by an elastic force

$$F\left(z,t\right)=-K_{\mathrm{pull}}\left[z-\left(z_{0}+\mathrm{vt}\right)\right]$$

from the cytoplasmic to the extracellular side. Here *z*, our chosen reaction coordinate, is position along the pore axis, *K*_*pull*_ = 2000 kJ mol^−1^ nm^−2^ is the stiffness of a harmonic spring one end of which moved with constant velocity *v* = 10 nm/ns (pull rate) along *z* while the center of mass of the calcein molecule was attached to the opposite spring end and also restrained to move along the pore axis.

For the actual umbrella sampling simulations, window centers were initially spaced at 2 Å and the whole simulated system underwent a short energy minimization process for each window. Thereafter we followed the MD trajectory, whereby the calcein center of mass was restrained by an elastic force

$$F_{\mathrm{umb},i}\left(z\right)=-K_{\mathrm{umb}}\left(z-z_{i}\right)$$

where *K*_*umb*_ = 1000 kJ mol^−1^ nm^−2^ is the elastic force constant and *z*_*i*_ is the position of the *i*–th window center along z axis. The dynamics was initially followed for 1 ns for each window. After obtaining a preliminary PMF profile, we refined the spacing to 1 Å and extended the duration of the simulated dynamics in the region were the PMFs in Figure 5 are rapidly increasing, until we reached convergence at 3.5 ns (meaning that the PMF profiles did not change appreciably by lengthening the simulation). The final total number of windows was *n* = 41 for both charged and uncharged calcein. Overall, the simulation time used for evaluating the two PMF profiles was in excess of 200 ns. All MD simulations were performed with Gromacs 4.5 software [29] using the Amber03 force field, in the NTV ensemble [26]. Temperature was kept constant at 300 K using the Berendsen thermostat [30]. Particle Mesh Ewald summation [31] was used for the long–range electrostatic interactions, with a cut off of 1.0 nm for the direct interactions. The simulation time step was comprised between 1 and 2 fs.

#### Estimate of transition rate from PMF

We assimilated the permeation process through a *complete* gap junction channel to overcoming a free energy profile, *W*(*z*), as described in Ref. [16]. We assumed *W*(*z*) to be a symmetric function obtained by reflecting the hemichannel PMF in Figure 2A about a vertical axis through the *z*_*H*_ abscissa corresponding to its extracellular end (see inset of Figure 5). Since both PMF peaks exceed by far the thermal energy (Figure 5), the transition rate can be derived by the liquid phase adaptation of the classical Eyring transition state theory [15,16,32]. We thus computed the transition rate, *k*, as

$$k=\alpha k_{0}e^{-\left[W\left(z_{M}\right)-W\left(z_{0}\right)\right]/k_{B}T}$$

where *z*_*M*_ and *z*_0_ are, respectively, the axial coordinate of the PMF maximum (in the extracellular vestibule) and minimum (in the cytoplasmic mouth of the channel; see Figure 2A,B). The prefactor *k*_0_ is given by

$$k_{0}=\sqrt{\frac{k_{B}T}{2\mathrm{\pi m}}}{\left[\int_{z_{0}}^{z_{M}}e^{-W\left(z\right)/k_{B}T}\mathrm{dz}\right]}^{-1}$$

where *m* is the mass of the calcein molecule. A molecule that starts from z_*M*_ descends down the *W*(*z*) profile pushed by a mean force

$$F=-\frac{\mathrm{dW}\left(z\right)}{\mathrm{dz}}$$

which favors its exit from the channel in either direction. This process is several orders of magnitude more probable than the reverse one, i.e. climbing *W*(*z*) in the uphill direction. For this reason we computed the above integral over the interval [z_0_, z_*M*_] considering that the rate–limiting step in the permeation process is determined by reaching coordinate *z*_*M*_, i.e. the apex of the free energy barrier.

The product:

$$k_{\mathrm{TST}}\equiv k_{0}e^{-\left[W\left(z_{M}\right)-W\left(z_{0}\right)\right]/k_{B}T}$$

is the classical transition state theory rate in liquid phase [15,16]. It represents the frequency with which the calcein molecules reaches *z*_*M*_ starting from z_0_.

A molecule that has reached *z*_*M*_ moves forward, towards the opposite end of the complete channel, with a certain probability represented by the positive factor *α* < 1, also known as transmission coefficient. To estimate *α* we used a Brownian Dynamics approach in the presence of the external mean force *F*; namely, we numerically solved the motion equation:

$$\mathrm{\Delta z}=F\frac{\mathrm{D\Delta t}}{k_{B}T}+\xi \sqrt{2\mathrm{D\Delta t}}$$

where ξ is a Gaussian white noise process, with zero mean and a time correlation function represented by Dirac’s delta function [33]; *D* = 9.2×10^–7^ cm^2^/s, the diffusion coefficient of calcein, was taken from Ref. [34] as this figure is in good agreement with the value we obtained after a 100 ns MD simulation in the bulk. In this mean–field–like approximation, the channel was not allowed to fluctuate and it interacted with the calcein molecule only through *F*, while thermal fluctuations due to the collision with water molecules were synthetically taken into account in the diffusion coefficient and the random forces ξ. The time step for the Brownian Dynamics simulation was set to 1 ps. This coarse−grained model allowed us to simulate 10^6^ transitions of a calcein molecule with reasonable use of CPU time. We then estimated the transmission coefficient *α* as the fraction of simulations that yielded a calcein molecule on the opposite side of the complete gap junction channel. Table 1 summarize the computed quantities used in this work.

**Table 1:** Summary of computed quantities

	***W*(*z***_***M***_**)(*k***_***B***_***T***)	***α***	***k*(s**^**−1**^**)**
Charged	45.2	0.01	7.7×10^–11^
Uncharged	19.6	0.16	352

Final note: the estimates of the transition rates represent the number of transitions (per unit time) that occur under saturating conditions, i.e. when the wait time between successive transitions is null. To realize such conditions, the bulk calcein concentration in cell 1 must be such that (at least) one calcein molecule is present, at any given time, within the cytoplasmic vestibule of the channel. To assess whether saturation was achieved under our experimental conditions (with a calcein concentration in the patch pipette equal to 125 μm), we simulated the diffusion of calcein inside the cell as a Brownian random walk. The results of this independent set of simulations indicate that the number of calcein molecules diffusing from bulk cytoplasm to the vestibule of an individual hemichannel is 2×10^4^ per second, suggesting that the zero wait state condition is a reasonable assumption.

## Abbreviations

CFP: Cyan Fluorescent Protein;CL: Cytoplasmatic loop;E1,E2: Extracelullar loop 1,2;hCx26wt: Wild type human connexin 26;hCx30wt: Wild type human connexin 30;ICS: Intra cellular solution;MD: Molecular dynamics;NT: N terminus;NTV–ensemble: Ensemble with fixed number of particles, volume and temperature;PMF: Potential of mean force;ROI: Region of interest;SEM: Standard error of the mean;TM1 to 4: Transmembrane helix 1 to 4

## Competing interests

The authors declare that they have no competing interests.

## Authors’ contributions

FZ Designed and performed simulations. Analyzed results the of simulations and drafted the manuscript. GP Designed and performed simulations. MFS Performed part of the experiments and simulations. MB Coordinated the experimental work and analyzed the results. FM Coordinated the work. Analyzed results, drafted and revised the manuscript. All authors read and approved the final manuscript.
